# Development and External Validation of a Postoperative Risk Score for Venous Thromboembolism in Epithelial Ovarian Cancer

**DOI:** 10.1002/kjm2.70223

**Published:** 2026-05-20

**Authors:** Qiu‐Lin Cui, Yu‐Shi He, Xuan‐Hui Wang, Cheng Li, Gang Wang, Ming Chen, Shu‐Min Chen

**Affiliations:** ^1^ Department of Obstetrics and Gynecology Sichuan Provincial Maternity and Child Health Care Hospital Chengdu City China; ^2^ Department of Obstetrics and Gynecology, The First Affiliated Hospital Sun Yat‐sen University Guangzhou City China; ^3^ Department of Obstetrics and Gynecology, The Seventh Affiliated Hospital Sun Yat‐sen University Shenzhen City China

**Keywords:** epithelial ovarian cancer, ovarian cancer, risk score, validation, venous thromboembolism

## Abstract

Venous thromboembolism (VTE), including deep vein thrombosis and pulmonary embolism, is a major complication in women with epithelial ovarian cancer (EOC). We aimed to develop and externally validate an early postoperative risk score to identify EOC patients at increased risk of VTE after debulking surgery. We retrospectively analyzed a development cohort (*N* = 358) to derive and internally validate a prediction model and an independent external cohort (*N* = 158) for external validation. Candidate clinical and laboratory variables were evaluated using least absolute shrinkage and selection operator (LASSO) and multivariable logistic regression. The final model was presented as a nomogram and converted into an integer‐based scoring system. Postoperative VTE within 30 days of surgery occurred in 12.01% of patients in the development cohort. Age, body mass index (BMI), preoperative international normalized ratio (INR), preoperative aspartate aminotransferase (AST), postoperative C‐reactive protein (CRP), and postoperative platelet count (PLT) were retained as predictors. The score incorporated age, BMI, INR, AST, CRP, and postoperative platelet count. Using a cutoff of 5 points, discrimination was high in the training and internal validation cohorts and acceptable in external validation (AUC 0.990 [95% CI 0.981–1.000], 0.934 [0.874–0.995], and 0.791 [0.653–0.929], respectively). In external validation, discrimination was comparable to the G‐Caprini score (AUC 0.759 [0.627–0.892]) with overlapping confidence intervals. Because the proposed score incorporates postoperative biomarkers, it is intended for early postoperative risk reassessment to support targeted surveillance and individualized post‐surgical prevention strategies, rather than preoperative prophylaxis decision‐making.

## 1 Introduction

Ovarian cancer remains the most lethal gynecologic malignancy, with an estimated 19,680 new cases and 12,740 deaths in 2024 in the United States. Importantly, epithelial ovarian, fallopian tube, and primary peritoneal carcinomas account for approximately 90% of ovarian malignancies [1]. Venous thromboembolism (VTE), including deep vein thrombosis and pulmonary embolism, is a common and potentially fatal complication in patients with cancer. Across malignancies, VTE incidence ranges from 4% to 20% [2] and is a leading cause of morbidity and mortality. Cancer‐associated thrombosis (CAT) is often cited as the second leading cause of death in patients with cancer after progression of the malignancy itself [3]. Tumor types such as pancreatic, brain, lung, primary hepatic, and ovarian cancers show particularly high thrombotic risk, and CAT is associated with increased mortality across tumor types [4, 5].

Patients with gynecologic cancers, especially epithelial ovarian cancer (EOC), are at increased risk of VTE due to patient factors (older age and comorbidities), tumor burden (advanced stage, bulky pelvic disease, ascites), and treatment‐related factors (extensive cytoreductive surgery, chemotherapy exposure, prolonged operative time, and immobility) [6, 7, 8]. In addition, systemic inflammation contributes to thrombogenesis, and inflammatory biomarkers such as C‐reactive protein (CRP) and composite indices have been associated with VTE risk in cancer populations [9, 10, 11].

Several risk assessment tools, including Caprini‐based models, are used to stratify perioperative VTE risk and guide thromboprophylaxis. However, these tools are largely designed for preoperative assessment and may not capture dynamic changes in coagulation and inflammation after major oncologic surgery. In ovarian cancer, thrombotic risk may evolve rapidly after surgery because endothelial injury, immobility, and postoperative inflammation peak in the early postoperative period. Therefore, an early postoperative reassessment tool may complement preoperative models by identifying patients who warrant intensified surveillance (e.g., early ultrasound/CT for suspected cases) and individualized continuation or escalation of postoperative prophylaxis according to bleeding risk and institutional practice.

In this retrospective study, we aimed to identify predictors of postoperative VTE within 30 days after debulking surgery in patients with EOC, develop a prediction model incorporating clinical and perioperative laboratory variables, and translate the model into an integer‐based scoring system for clinical use. We further evaluated model performance through internal validation and external validation in an independent cohort.

## 2 Methods

### 2.1 Patients

This retrospective cohort study was conducted in accordance with the Declaration of Helsinki and approved by the Ethics Committee of The First Affiliated Hospital of Sun Yat‐sen University (No. S/55904). Given the retrospective design and use of de‐identified data, the requirement for informed consent was waived.

The study consisted of two stages: model development and external validation. The development cohort included consecutive patients treated at The First Affiliated Hospital of Sun Yat‐sen University between January 2018 and December 2021. Patients from Sun Yat‐sen Memorial Hospital, Sun Yat‐sen University, during the same period were used as an independent external validation cohort. Eligible patients were women who (i) underwent primary debulking surgery and (ii) had a postoperative histopathological diagnosis of EOC confirmed by at least two pathologists.

**FIGURE 1:**
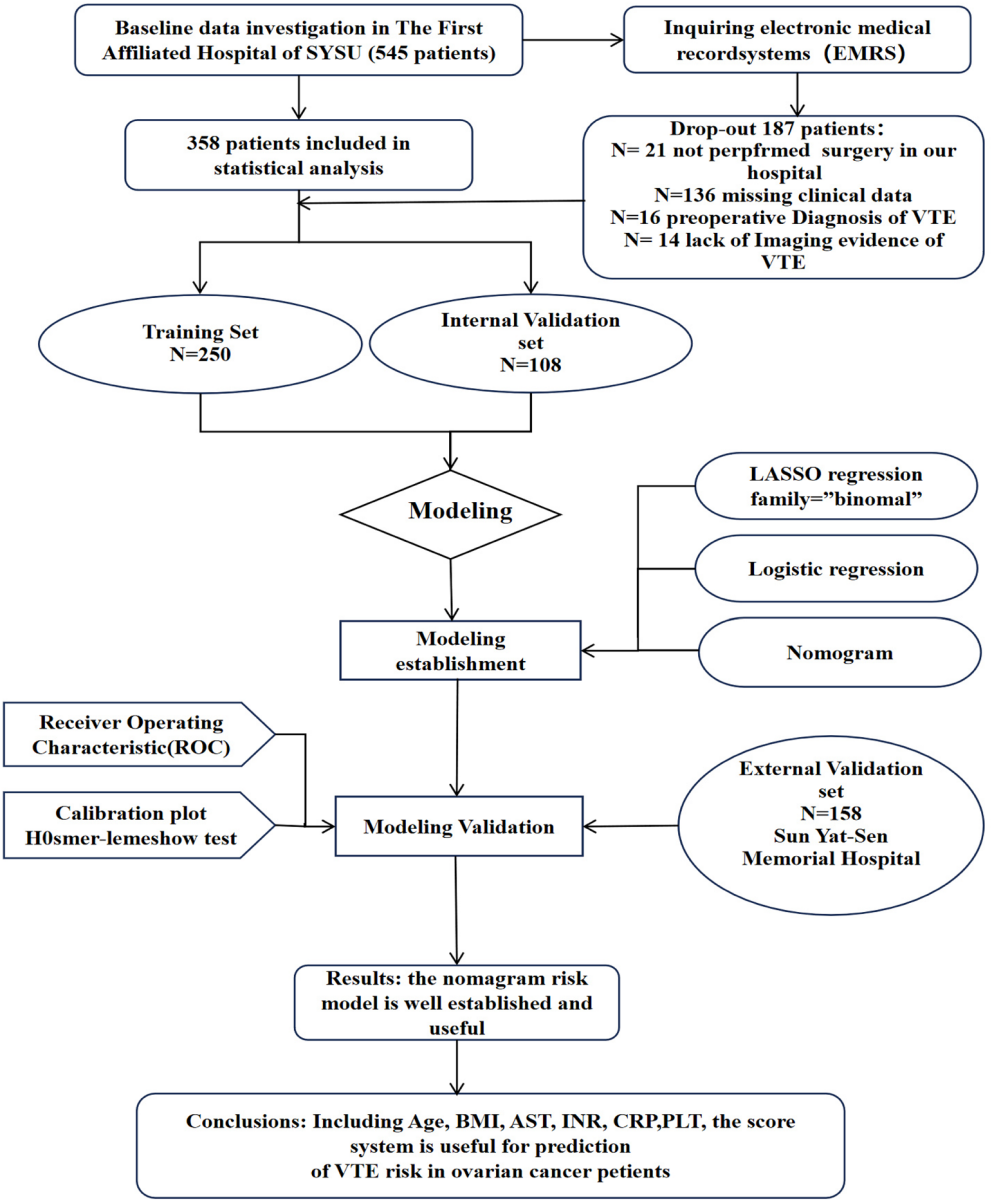
Flow diagram of the study design. Abbreviations: BMI, body mass index; AST, aspartate transaminase; INR, international normalized ratio; CRP, C‐reactive protein; PLT, platelet count.

The primary outcome was postoperative venous thromboembolism (VTE) occurring within 30 days after surgery, confirmed by imaging (compression ultrasonography for deep vein thrombosis and/or computed tomography pulmonary angiography for pulmonary embolism). Patients with documented preoperative VTE, unclear thrombotic etiology, missing key predictor or outcome data, or loss to follow‐up within 30 days were excluded. The development cohort was randomly divided into a training set (70%) and an internal validation set (30%) using a computer‐generated random sequence. Figure 1 shows patient inclusion/exclusion and the analytic workflow, including development of (i) a clinical model and (ii) an expanded clinical–inflammation model and derivation of the integer score.

### 2.2 Data Collection

Clinical data were extracted from electronic medical records (EMR), including demographic variables (age, gravidity, and body mass index), comorbidities (hypertension, diabetes), surgical variables (operative duration, blood loss, maximum tumor diameter, residual disease, and number of lymph nodes removed), tumor‐related characteristics (ascites volume), and laboratory parameters measured within 7 days preoperatively and within 24 h postoperatively. Optimal debulking surgery was defined as no macroscopic residual disease or residual disease < 1 cm. Ascites volume was categorized as mild (< 500 mL), moderate (500–1000 mL), or severe (> 1000 mL). Inflammatory indices such as NLR, PLR, and LMR were calculated from laboratory data. The G‐Caprini score was calculated using preoperative clinical variables according to the published Caprini risk assessment model (and gynecologic oncology adaptation, if applicable), and was used as a benchmark preoperative tool for discrimination comparison only.

### 2.3 Model Construction and Statistical Analysis

Continuous variables were summarized as mean ± standard deviation or median (interquartile range), and categorical variables as counts (percentages). Between‐group comparisons (VTE vs. non‐VTE) were performed using the *χ* ^2^ test or Fisher's exact test for categorical variables and Student's *t*‐test or the Mann–Whitney *U* test for continuous variables, as appropriate.

Prediction modeling was conducted in the training set. Candidate predictors were prespecified based on clinical plausibility, prior literature, and data availability. To reduce overfitting, least absolute shrinkage and selection operator (LASSO) logistic regression was applied, with the optimal penalty parameter (*λ*) selected using 10‐fold cross‐validation. Variables with non‐zero coefficients were entered into multivariable logistic regression to construct the final model. No univariate ROC‐ or AUC‐based pre‐screening was performed.

In the development cohort, 43 postoperative VTE events occurred, with approximately 30 events in the training set after random allocation (70%). The final model retained six predictors, yielding an events‐per‐variable (EPV) ratio of approximately 5 in the training cohort. Although lower than the traditional rule‐of‐thumb of 10, penalized regression with shrinkage was used to mitigate overfitting and improve model stability.

Regression coefficients from the final multivariable model were used to construct a nomogram and were proportionally transformed into an integer‐based scoring system using identical variable definitions and coding. The predicted probability of postoperative VTE was calculated using the logit transformation of the final model equation.

Model discrimination was evaluated using the area under the receiver operating characteristic curve (AUC) in the training, internal validation, and external validation cohorts. Calibration was assessed using calibration plots generated with the rms package in R, and calibration‐in‐the‐large and calibration slope were reported. Bootstrap internal validation (1000 resamples) was performed in the training cohort to estimate optimism‐corrected performance.

The optimal cutoff for the scoring system was determined using the Youden index in the training set. Sensitivity, specificity, positive likelihood ratio (PLR), and negative likelihood ratio (NLR) were calculated with 95% confidence intervals (CIs). CIs for sensitivity and specificity were estimated using exact binomial methods, and CIs for PLR and NLR were calculated on the logarithmic scale. Missing data were evaluated for extent and pattern. As missingness was < 5% for all predictors retained in the final model, complete‐case analysis was performed. No synthetic resampling or class‐weight adjustment was applied. Statistical analyses were conducted using SPSS version 25.0 (IBM Corp., Armonk, NY, USA) and R version 4.4.1. All tests were two‐sided, and *p* < 0.05 was considered statistically significant.

Survival analyses (progression‐free survival and overall survival) were performed as exploratory analyses to describe the prognostic association of postoperative VTE and were not used for model development or score derivation.

## 3 Results

### 3.1 Characteristics of the Study Cohort

A total of 358 consecutive patients with EOC who underwent primary debulking surgery at The First Affiliated Hospital of Sun Yat‐sen University were included in the development cohort. Postoperative VTE within 30 days occurred in 43 patients (12.01%). The cohort was randomly divided into a training set (*n* = 250, 70%) and an internal validation set (*n* = 108, 30%). An independent external validation cohort consisted of 158 patients treated at Sun Yat‐sen Memorial Hospital during the same period. The detailed study flow is presented in Figure 1. Given the VTE incidence of 12.01%, no synthetic resampling or class‐weight adjustment was applied. Model performance was interpreted considering outcome prevalence.

Baseline demographic, clinical, and laboratory characteristics stratified by VTE status are summarized in Table 1. Comparisons of inflammatory indices between VTE and non‐VTE groups are presented in Table 2. Preoperative variables were defined as values obtained within 7 days before surgery, and postoperative laboratory variables were defined as values obtained within 24 h after surgery.

**TABLE 1:** Clinical characteristics of ovarian cancer patients during perioperative period.

	VTE group(*n* = 43)	N‐VTE group(*n* = 315)	Value	*p*	Training set (*n* = 250)	Internal Validation set (*n* = 108)	Value	*p*	External Validation set (*n* = 158)
Age^a^ (years)	59.77 ± 11.02	51.85 ± 11.22	*t =* 4.345	**< 0.0001**	53.13 ± 10.33	52.04 ± 12.5	*t* = 1.208	0.2278	54.78 ± 10.65
BMI (kg/m^2^)									
< 24.0	20 (46.51%)	273 (86.67%)	*χ* ^***2***^ **=** 38.40	**< 0.0001**	203 (81.2%)	90 (83.33%)	*χ* ^*2*^ = 0.2310	0.6308	130 (82.28%)
≥ 24.0	23 (53.49%)	42 (13.33%)			47 (18.8%)	18 (16.67%)			28 (17.72%)
Gravidity									
< 2	17 (39.53%)	83 (26.35%)	*χ* ^***2***^ **=** 3.268	0.0707	67 (26.8%)	33 (30.56%)	*χ* ^*2*^ = 0.3583	0.5494	94 (59.49%)
≥ 2	26 (60.47%)	232 (73.65%)			183 (73.2%)	75 (69.44%)			64 (40.51%)
Hypertension									
No	31 (72.09%)	261 (82.86%)	*χ* ^***2***^ **=** 2.915	0.2877	203 (81.2%)	89 (82.41%)	*χ* ^*2*^ = 0.01487	0.9030	128 (81.01%)
Yes	12 (27.91%)	54 (17.14%)			47 (18.8%%)	19 (17.59%)			30 (18.99%)
Diabetes									
No	38 (88.37%)	291 (92.38%)	*χ* ^***2***^ **=** 0.8168	0.3701	232 (92.8%)	97 (89.81%)	*χ* ^*2*^ = 0.5463	0.4598	145 (91.77%)
Yes	5 (11.63%)	24 (7.62%)			18 (7.2%)	11 (10.19%)			13 (8.23%)
History of thrombosis									
No	42 (97.67%)	314 (99.68%)	/	0.2261	249 (99.6%)	107 (99.07%)	/	0.5129	150 (94.94%)
Yes	1 (2.33%)	1 (00.32%)			1 (0.4%)	1 (0.93%)			8 (5.06%)
History of Cancer^c^									
No	35 (86.05%)	290 (92.06%)	/	**0.0426**	231 (92.4%)	94 (87.04%)	*χ* ^*2*^ = 1.991	0.1582	153 (96.84%)
Yes	8 (13.95%)	25 (7.94)			19 (7.6%)	14 (12.96%)			5 (3.16%)
G‐Caprini score									
Before operation									
≤ 2	3 (6.98%)	37 (11.75%)	*χ* ^2^ = 0.9321	0.6275	29 (11.6%)	11 (10.2%)	*χ* ^2^ = 2.015	0.3651	11 (7%)
3–4	39 (90.70%)	269 (85.40%)			216 (86.4%)	92 (85.2%)			142 (89.8%)
≥ 5	1 (2.32%)	9 (28.57%)			5 (2%)	5 (4.6%)			5 (3.2)
Ascites									
< 500	27 (62.79%)	249 (79.05%)	*χ* ^2^ = 7.927	**0.0190**	191 (76.4%)	85 (78.7%)	*χ* ^2^ = 0.2745	0.8718	119 (75.3%)
500–1000	2 (4.65%)	18 (5.71%)			14 (5.6%)	6 (55.6%)			5 (3.2%)
≥ 1000	14 (32.56%)	48 (15.24%)			45 (18%)	17 (15.7%)			33 (20.9%)
Blood loss^b^ (mL)	500 (300–1000)	400 (200–800)	*W* = 5259	**0.0166**	400 (200–800)	500 (200–800)	*W* = 11,759	0.1422	200 (100–400)
Duration of surgery^a^	384 (292.5–446.5)	315 (251–402.5)	*W* = 5164	**0.0112**	330.4 ± 130.7	360.8 ± 128.4	*t* = 2.026	**0.0435**	250.9 ± 102.3
Maximum tumor diameter^b^ (cm)	11 (7–14)	7.00 (0.50 ~ 30.00)	*W* = 4302	**< 0.0001**	7.1 (5–12)	7 (4–11)	W = 12,708	0.3776	6 (4–10.75)
Number of lymph nodes removed									
< 20	13 (39.39%)	140 (46.98%)	/	0.4644	104 (45.6%)	49 (47.6%)	/	0.8119	36 (48.6%)
≥ 20	20 (60.61%)	158 (53.02%)			124 (54.4%)	54 (52.4%)			38 (51.4%)
Residual disease (cm)									
< 1	39 (90.7%)	297 (95.29%)	/	**< 0.0001**	232 (92.8%)	104 (96.3%)	/	0.24	137 (86.7%)
≥ 1	4 (9.3%)	18 (5.71%)			18 (7.2%)	4 (3.7%)			21 (13.3%)
Histology									
Serous carcinoma	23 (53.49%)	222 (70.48%)	*χ* ^***2***^ **=** 42.159	**< 0.0001**	163 (65.3%)	82 (75.93%)	*χ* ^*2*^ = 5.314	0.2566	130 (82.28%)
Mucinous carcinoma	2 (4.65%)	16 (5.1%)			43 (17.2%)	11 (10.19%)			7 (4.43%)
Clear cell carcinoma	13 (30.23%)	42 (13.33%)			24 (9.6%)	10 (9.26%)			10 (6.33%)
Endometroid carcinoma	2 (4.65%)	32 (10.1%)			15 (6%)	3 (2.78%)			5 (3.16%)
Mix carcinoma	3 (6.98%)	3 (0.95%)			4 (1.6%)	2 (1.85%)			6 (3.8%)
FIGO stage									
I	9 (20.93%)	71 (22.54%)	*χ* ^***2***^ **=** 3.726	0.2926	62 (24.8%)	18 (16.67%)	*χ* ^*2*^ = 3.142	0.3702	19 (12.03%)
II	2 (4.65%)	34 (10.79%)			24 (9.6%)	12 (11.11%)			9 (5.7%)
III	20 (46.51%)	155 (49.21%)			117 (46.8%)	58 (23.15%)			94 (59.5%)
IV	12 (29.71%)	55 (17.46)			47 (18.8%)	20 (18.52%)			36 (22.78%)

*Note:* Values significant at *p* < 0.05 are shown in bold.

Abbreviations: BMI, Body mass index; FIGO, Federation International of Gynecology and Obstetrics; VTE, Venous thromboembolism.

^a^ Mean ± standard deviation, range.

^b^ Median, interquartile range (IQR), range.

^c^ Breast cancer (*n* = 10), lung cancer (*n* = 2), bowel cancer (*n* = 9), thyroid cancer (*n* = 3); pancreatic neuroendocrine tumors (*n* = 1).

**TABLE 2:** Inflammatory factors of ovarian cancer patients before and after operation.

	VTE	N‐VTE	Value	*p*
CRP^a^ (mg/L)				
Post‐operation	83.2 (50.46–111.25)	65.68 (40.92–96.66)	*W* = 5176	**0.0438**
WBC^a^ (10*9/L)				
Pre‐operation	6.994 (6.05–8.115)	6.19 (5.21–7.52)	*W* = 5071	**0.0442**
Post‐operation	11.15 (8.145–13.26)	11.32 (8.97–14.12)	*W* = 13,266	**< 0.0001**
Neut^a^ (10*9/L)				
Pre‐operation	4.34 (3.78–5.51)	3.95 (3.01–5.16)	*W* = 5410	0.1460
Post‐operation	8.91 (6.84–10.855)	9.77 (7.61–12.805)	*W* = 5627	0.0718
Mono^a^ (10*9/L)				
Pre‐operation	0.5 (0.415–0.62)	0.47 (0.38–0.59)	*W* = 5625	0.2704
Post‐operation	0.49 (0.325–0.725)	0.51 (0.36–0.67)	*W* = 6543	0.7196
Lym^a^ (10*9/L)				
Pre‐operation	1.533 (1.19–1.935)	1.53 (1.175–1.85)	*W* = 6291	0.988
Post‐operation	0.83 (0.635–1.125)	0.74 (0.49–1.07)	*W* = 5866	0.1548
Plt^a^ (10*9/L)				
Pre‐operation	305 (228–342.5)	266 (205–334.5)	*W* = 6190	0.8574
Post‐operation	227 (176–286)	219 (173.5–286.5)	*t* = 0.7105	0.4779
NLR^a^				
Pre‐operation	3.005 (1.985–4.70)	2.65 (1.788–4.168)	*W* = 6124	0.3096
Post‐operation	10.043 (7.262–15.52)	13.707 (8.373–21.54)	*W* = 5578	0.0605
PLR^a^				
Pre‐operation	195.513 (139.85–298.27)	172.38 (127.66–269.163)	*W* = 5943	0.1934
Post‐operation	265.421 (203.226–414.032)	298.936 (204.91–419.231)	*W* = 6363	0.5211
LMR^a^				
Pre‐operation	3 (1.983–4.117)	3.1365 (2.356–4.167)	*W* = 6369	0.5557
Post‐operation	1.6 (1.177–2.53)	1.447 (1.238–2.094)		
SII^a^				
Pre‐operation	931.62 (564.95–1418.21)	604.297 (403.84–1215.957)	*W* = 5638	0.0747
Post‐operation	2679.47 (1531.5–3656.12)	2900.6 (1646.77–4854.149)	*W* = 6033	0.2426
Alb^a^ (g/L)				
Pre‐operation	35.35 (31.75–38.75)	38 (35.05–40.05)	*W* = 4542	**0.0038**
Post‐operation	29 (25–34)	27 (23.7–30)	*W* = 5574	0.0630
HB^b^ (g/L)				
Pre‐operation	112.725 (100.5–122)	113 (102–112)	*W* = 6670	0.8722
Post‐operation	101 (89–114)	112 (94–116)	*t* = 0.7105	0.4779
PT^a^ (s)				
Pre‐operation	11.55 (11.1–12.15)	11.5 (11–12)	*W* = 5788	0.4035
Post‐operation	13.1 (11.9–14.3)	11.9 (11.2–12.8)	*W* = 4157	**0.0002**
APTT^a^ (s)				
Pre‐operation	26.7 (25.4–28.25)	27.3 (25.8–29.75)	*W* = 5640	0.2816
Post‐operation	28.7 (27.1–35.3)	27.7 (25.4–30.8)	*W* = 4898	**0.0115**
FIB^a^ (g/L)				
Pre‐operation	3.515 (3.023–4.2675)	3.11 (2.6–3.905)	*W* = 4885	**0.0203**
Post‐operation	3.6 (2.83–4.63)	3.74 (2.92–4.48)	*W* = 5835	0.8216
INR^a^				
Pre‐operation	1.02 (0.968–1.08)	0.98 (0.94–1.03)	*W* = 4813	**0.0145**
Post‐operation	1.15 (1.01–1.2)	1.02 (0.96–1.07)	*W* = 3967	**0.0001**

*Note:* Values significant at *p* < 0.05 are shown in bold.

Abbreviations: Alb: Albumin; APTT: Activated partial thromboplastin time; CRP: C‐reactive protein; FIB: Fibrinogen; HB: Hemoglobin; INR: International normalized ratio; LMR: lymphocyte to monocyte ratio; Lym: Lymphocyte; Mono: Monocytes; Neut: neutrophil; NLR: Neutrophil to lymphocyte ratio; PLR: Plated to lymphocyte ratio; Plt: Platelet; PT: Prothrombin time; SII: Systemic immune‐inflammation index; VTE: Venous thromboembolism; WBC: White blood cell.

^a^ Median, interquartile range (IQR), range.

^b^ Mean ± standard deviation, range.

Cox regression analysis showed that patients who developed postoperative VTE had significantly worse PFS and OS compared with those without VTE (*p* < 0.001). This survival analysis was exploratory and intended to describe the prognostic association of VTE; the prediction endpoint for model development remained postoperative VTE within 30 days.

### 3.2 Construction of a Clinical Model in the Training Set

**FIGURE 2:**
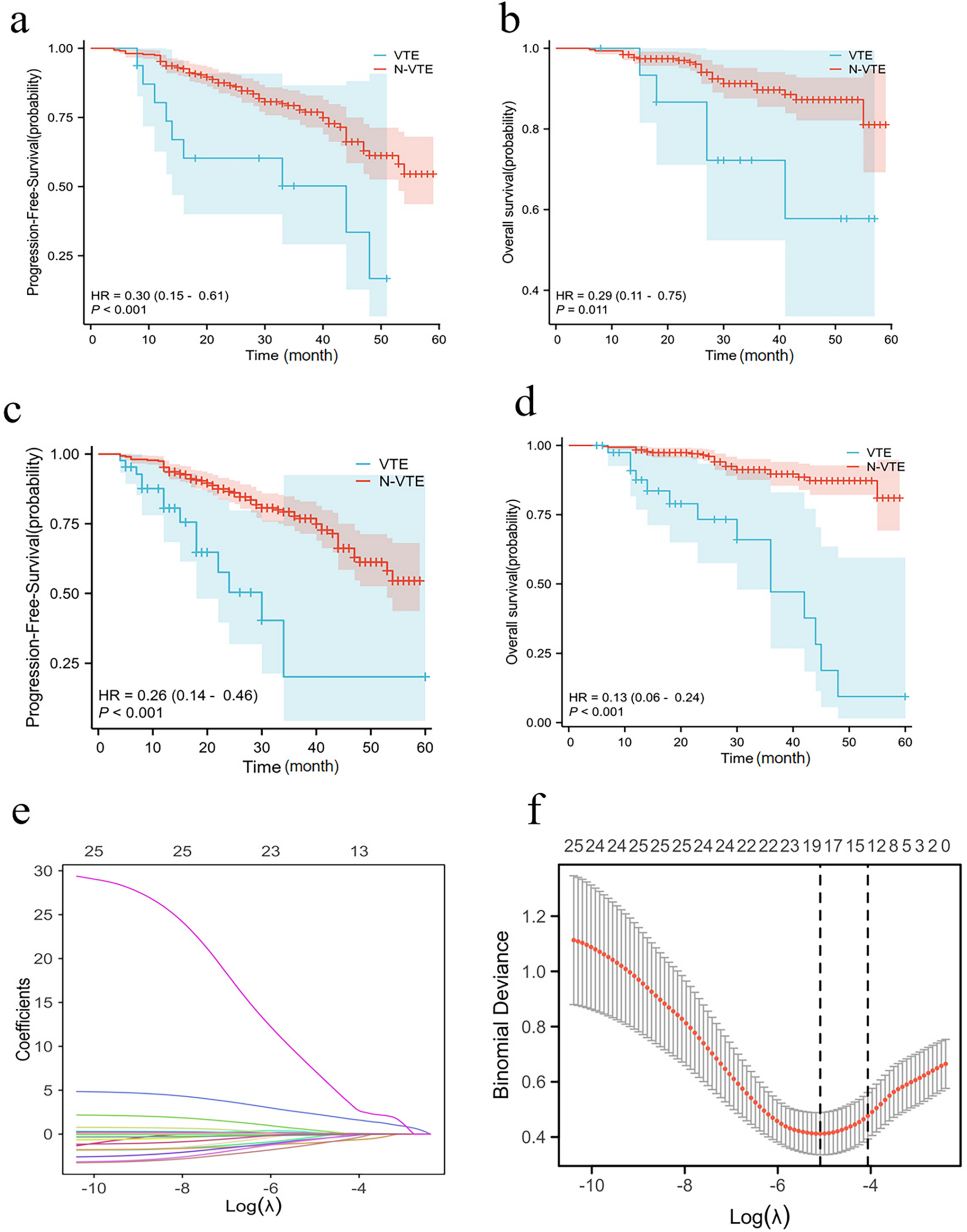
Association between thromboembolic events and survival outcomes in patients with epithelial ovarian cancer (EOC), and LASSO variable selection in the training set. (a, b) Cox regression analysis of progression‐free survival (PFS) and overall survival (OS) according to the presence or absence of thromboembolic events before surgery. (c, d) Cox regression analysis of PFS and OS according to the presence or absence of thromboembolic events after surgery. (e) Coefficient profile plot from LASSO binary logistic regression in the training set, showing variable shrinkage across the log(λ) sequence. Eighteen variables with non‐zero coefficients were retained at the optimal λ. (f) Selection of the optimal penalty parameter (λ) in the LASSO model using 10‐fold cross‐validation, plotted as binomial deviance versus log(λ).

**FIGURE 3:**
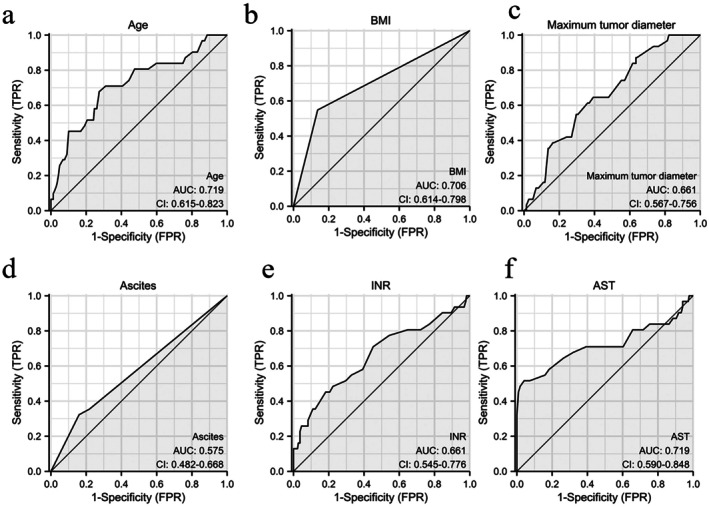
Receiver operating characteristic (ROC) curves of selected candidate predictors for postoperative VTE. (a) Age; (b) BMI; (c) maximum tumor diameter; (d) ascites volume; (e) INR; (f) AST. Abbreviations: BMI, body mass index; AST, aspartate transaminase; INR, international normalized ratio.

**FIGURE 4:**
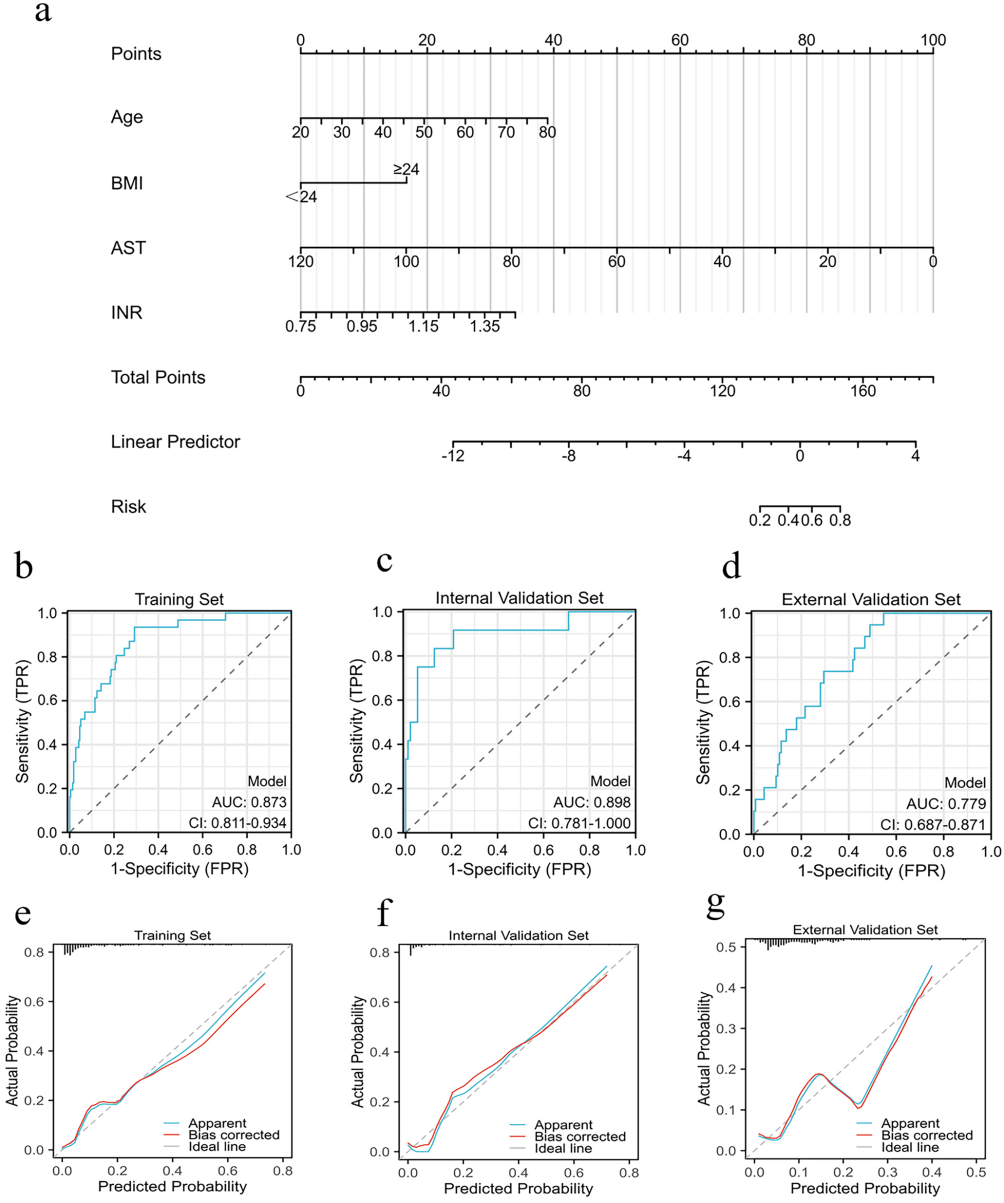
Nomogram and performance of the clinical prediction model. (a) Nomogram incorporating age, BMI, AST, and INR for individualized prediction of postoperative VTE. (b–d) ROC curves of the clinical model in the training set (b), internal validation set (c), and external validation set (d). (e–g) Calibration curves of the clinical model in the training set (e), internal validation set (f), and external validation set (g).

In the training cohort, LASSO logistic regression was applied to select candidate predictors from the predefined clinical and laboratory variables (Figure 2a,d). Eighteen variables were retained based on non‐zero coefficients (Figure 2e,f) and were subsequently entered into multivariable logistic regression analysis (Figure 3a–f). Four variables remained independently associated with postoperative VTE: age, BMI, preoperative AST, and preoperative INR (Figure 3a–f; Table 3). These predictors were used to construct a nomogram for individualized risk estimation (Figure 4a).

**TABLE 3:** Univariate and multivariate analysis of clinical factors associated with VTE after operation.

Characteristics	Total (*N*)	Univariate analysis		Multivariate analysis	
		Odds ratio (95% CI)	*p*	Odds ratio (95% CI)	*p*
Age	250	1.079 (1.040–1.120)	**< 0.001**	1.085 (1.038–1.134)	**< 0.001**
Number of lymph nodes removed	228				
≥ 20	124	Reference			
< 20	104	0.908 (0.381–2.166)	0.828		
BMI	250				
< 24	203	Reference		Reference	
≥ 24	47	7.650 (3.419–17.118)	**< 0.001**	8.268 (3.110–21.984)	**< 0.001**
Hypertension	250				
No	47	Reference			
Yes	203	0.513 (0.219–1.202)	0.124		
Diabetes	250				
No	232	Reference			
Yes	18	1.457 (0.397–5.352)	0.571		
Gravidity	250				
**<** 2	183	Reference			
≥ 2	67	1.601 (0.722–3.548)	0.247		
Maximum tumor diameter	250	1.095 (1.022–1.173)	**0.010**	1.080 (0.988–1.180)	0.089
Ascites	250				
**<** 500	191	Reference		Reference	
500–1000	14	0.658 (0.082–5.296)	0.694		
≥ 1000	45	2.443 (1.053–5.668)	**0.038**	0.161 (0.008–3.350)	0.238
Surgical methods	250				
Laparoscopy	40	Reference			
Laparotomy	210	0.527 (0.152–1.825)	0.312		
Duration of surgery	250	1.001 (0.999–1.004)	0.369		
Blood loss	250	1.000 (1.000–1.001)	0.122		
HE4	250	1.000 (0.999–1.001)	0.690		
AST	250	0.901 (0.843–0.964)	**0.003**	0.917 (0.859–0.979)	**0.009**
ALT	250	1.016 (0.995–1.037)	0.128		
PT	250	1.278 (0.897–1.819)	0.174		
APTT	250	1.042 (0.990–1.096)	0.112		
INR	250	1408.356 (31.789–62394.4038)	**< 0.001**	108.549 (1.022–11525.3094)	**0.049**

*Note:* Values significant at *p* < 0.05 are shown in bold.

Abbreviations: 95% CI, confidence intervals; ALT, alanine aminotransferase; APTT, activated partial thromboplastin time; AST, aspartate transaminase; BMI, body mass index; FIB, fibrinogen; HE4, human epididymis protein 4; INR, International normalized ratio; OR, odds ratio; PT, prothrombin time; VTE, venous thromboembolism.

The clinical model demonstrated good discrimination, with AUCs of 0.873 (95% CI 0.811–0.934) in the training set, 0.898 (95% CI 0.781–1.000) in the internal validation set, and 0.779 (95% CI 0.687–0.871) in the external validation cohort (Figure 4b–d). Calibration plots showed acceptable agreement between predicted and observed probabilities across datasets (Figure 4e–g).

These results support the feasibility of a clinical‐only model, but also suggest that performance may vary across centers, motivating evaluation of early postoperative biomarkers.

### 3.3 Construction of a Clinical Model With Inflammation‐Related Factors and Novel Scoring System in the Training Set

**FIGURE 5:**
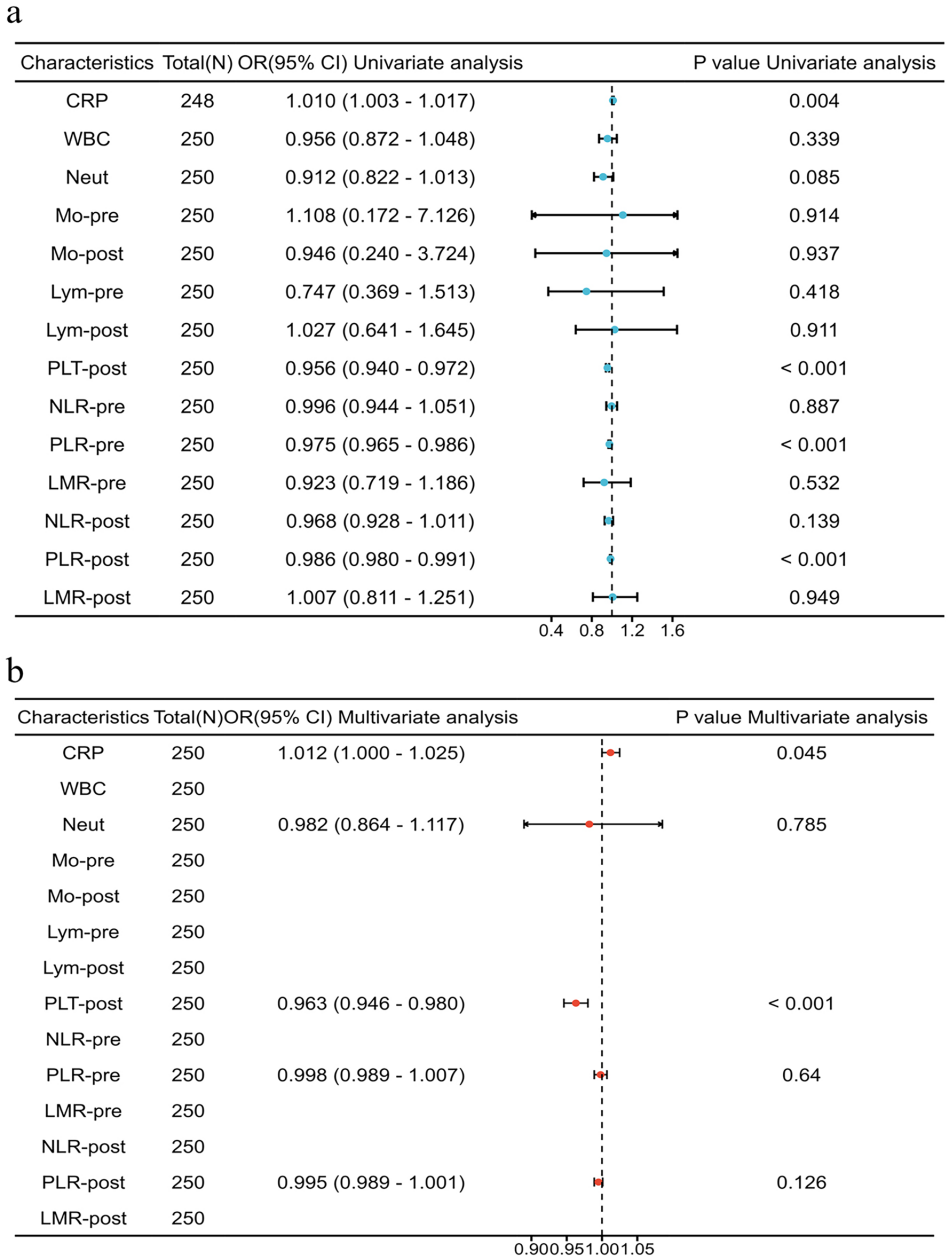
Univariate and multivariate analyses of inflammatory factors associated with postoperative VTE during the perioperative period. (a) Forest plot of univariate analysis results. (b) Forest plot of multivariate analysis results. Abbreviations: CRP, C‐reactive protein; WBC, white blood cell count; Neut, neutrophil; Mono, monocyte; Lym, lymphocyte; PLT, platelet count; NLR, neutrophil‐to‐lymphocyte ratio; PLR, platelet‐to‐lymphocyte ratio; LMR, lymphocyte‐to‐monocyte ratio.

Given the established role of inflammation in thrombogenesis, postoperative inflammatory parameters were further evaluated. Based on clinical rationale and availability within 24 h after surgery, postoperative CRP and platelet count (PLT) were prioritized among inflammatory candidates. In univariable analyses, postoperative CRP and PLT were significantly associated with postoperative VTE (Figure 5a,b). After multivariable adjustment, both CRP and PLT remained independently associated with postoperative VTE.

**FIGURE 6:**
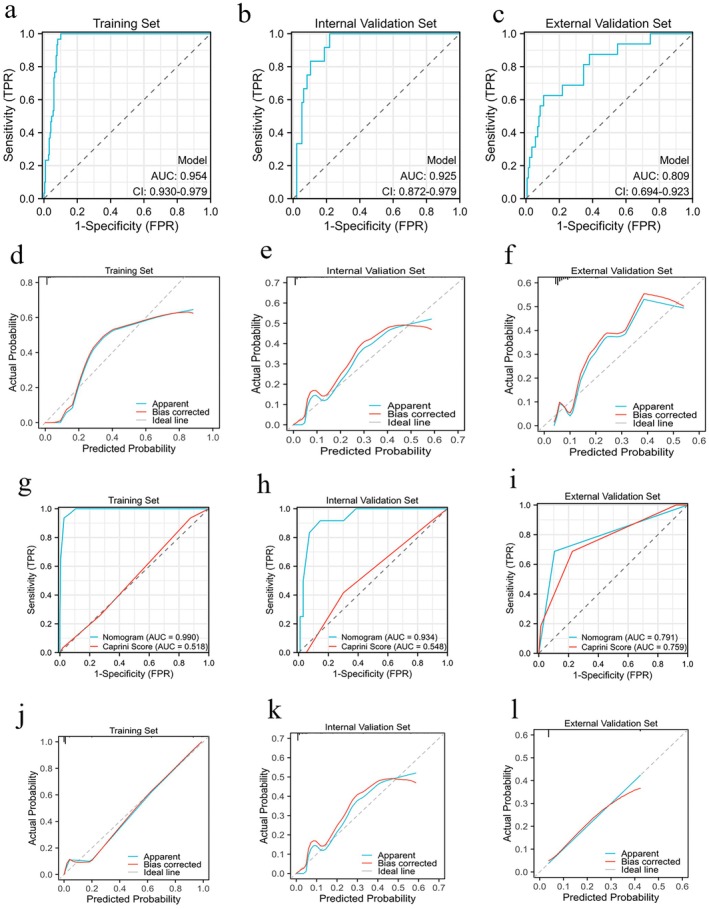
Discrimination and calibration of the clinical–inflammatory model and the derived scoring system. (a–c) ROC curves of the clinical–inflammatory model in the training set (a), internal validation set (b), and external validation set (c). (d–f) Calibration curves of the clinical–inflammatory model in the training set (d), internal validation set (e), and external validation set (f). (g–i) ROC curves comparing the new scoring system with the traditional G‐Caprini scoring system in the training set (g), internal validation set (h), and external validation set (i). (j–l) Calibration curves of the scoring system in the training set (j), internal validation set (k), and external validation set (l).

These inflammatory variables were integrated into the clinical model to construct an expanded model. The addition of CRP and PLT improved model discrimination in the development cohort. The AUC increased to 0.954 (95% CI 0.930–0.979) in the training set and 0.925 (95% CI 0.872–0.979) in the internal validation set. In the external validation cohort, the AUC was 0.809 (95% CI 0.694–0.923) (Figure 6a–c). Calibration remained acceptable in all cohorts (Figure 6d–f).

To facilitate clinical implementation, the nomogram was converted into an integer‐based scoring system according to regression coefficients (Table 4). The final score included age, BMI, AST, INR, CRP, and postoperative PLT, with weighted point assignments proportional to their effect sizes. The score definition was applied consistently across the training, internal validation, and external validation cohorts.

**TABLE 4:** A novel scoring system developed from multivariate analysis of the training set.

Parameters	Score generated from multivariate analysis (points)	Score modified from multivariate analysis (points)
Age (> 56 years)	0.0814	1
BMI (≥ 24)	2.1124	2
AST (≤ 12.5)	−0.0868	1
INR (≥ 1.055)	4.6872	5
CRP (≥ 74.53)	0.0124	1
PLT (≤ 129)	−0.0375	1

Abbreviations: AST, aspartate transaminase; BMI, body mass index; CRP, C‐reactive protein; INR, international standard ratio; PLT, platelet count.

### 3.4 Diagnostic Performance of the Scoring System Compared With Traditional G‐Caprini Score

Using the predefined cutoff, the scoring system demonstrated strong discrimination in the training and internal validation sets, with AUCs of 0.990 (95% CI 0.981–1.000) and 0.934 (95% CI 0.874–0.995), respectively. In the external validation cohort, the AUC was 0.791 (95% CI 0.653–0.929).

For comparison, the G‐Caprini score yielded AUCs of 0.518 (95% CI 0.414–0.623) in the training set, 0.548 (95% CI 0.377–0.719) in the internal validation set, and 0.759 (95% CI 0.627–0.892) in the external validation cohort (Figure 6g–i). Meanwhile, Figure 6j–l demonstrates well‐calibrated differential diagnosis of ovarian cancer patients with VTE across all three datasets.

Because the proposed score incorporates postoperative laboratory values, comparisons with preoperative tools such as G‐Caprini should be interpreted cautiously, as they represent different clinical time points. In the external cohort, discrimination was comparable and confidence intervals overlapped.

Additional diagnostic performance metrics, including sensitivity, specificity, PLR, and NLR, are presented in Table 5. In the external cohort, PLR and NLR are reported alongside AUC to provide complementary information on rule‐in and rule‐out performance.

**TABLE 5:** Diagnostic performance of the scoring system compared with G‐Caprini score in ovary cancer patients after surgery in three data sets.

Variables	Scoring system			G‐Caprini score		
AUC (95% CI)	Training set (*n* = 250)	Internal validation set (*n* = 108)	External validation set (*n* = 158)	Training set (*n* = 250)	Internal validation set (*n* = 108)	External validation set (*n* = 158)
AUC (95% CI)	0.990 (0.981–1.000)	0.934 (0.874–0.995)	0.791 (0.653–0.929)	0.518 (0.414–0.623)	0.548 (0.377–0.719)	0.759 (0.627–0.892)
Sensitivity % (95% CI)	71.9 (56.3–84.7)	61.1 (42.2–77.6)	56.3 (37.7–73.6)	44.5 (29.3–60.6)	46.7 (28.3–65.7)	57.5 (38.8–74.5)
Specificity % (95% CI)	78.8 (72.7–84.1)	74.4 (64.9–82.3)	63.1 (54.3–71.2)	56.7 (49.8–63.4)	55.0 (45.0–64.7)	56.8 (48.1–65.1)
PLR (95% CI)	3.39 (2.30–5.00)	2.39 (1.45–3.96)	1.53 (1.05–2.22)	1.03 (0.72–1.47)	1.04 (0.67–1.61)	1.33 (0.90–1.97)
NLR (95% CI)	0.36 (0.22–0.59)	0.52 (0.34–0.79)	0.69 (0.46–1.03)	0.98 (0.76–1.27)	0.97 (0.73–1.30)	0.75 (0.52–1.07)

Abbreviations: AUC, area under the curve; CI, confidence interval; NLR, negative likelihood ratio; PLR, positive likelihood ratio.

## 4 Discussion

In this retrospective cohort study, we developed and externally validated an early postoperative risk prediction model for VTE occurring within 30 days after primary debulking surgery in patients with EOC. Postoperative VTE occurred in 12.01% of patients in the development cohort and was significantly associated with worse PFS and OS. The final model incorporated routinely available variables and demonstrated good discrimination in the development and internal validation cohorts, with acceptable performance in the external validation cohort. Importantly, the simplified integer‐based scoring system preserved most of the discriminative ability of the full nomogram, supporting its potential clinical usability.

The primary clinical value of this score is not to replace preoperative risk assessment, but to provide early postoperative risk reassessment when inflammation and coagulation activation are most dynamic. In practice, the score may support targeted surveillance for occult VTE and inform individualized continuation or escalation of postoperative prophylaxis, depending on bleeding risk and institutional protocols. Our observed 30‐day postoperative VTE incidence is consistent with previously reported rates in ovarian cancer populations. VTE is a clinically significant complication that adversely affects survival, prolongs hospitalization, and increases healthcare costs [5, 12, 13, 14, 15]. The high thrombotic risk in ovarian cancer reflects a complex interplay between tumor biology, surgical trauma, and systemic inflammation.

Reasons include: thrombocytosis or hyperfibrinogenemia complicating 20%–40% of postoperative ovarian cancer patients, suggesting hypercoagulable blood [16]; vascular aberrations in ovarian tumor tissues leading to hypoxia, inadequate plasma component uptake by cancer cells, exacerbating ectopic coagulation factor and tissue factor expression [17], advanced age, advanced stages, larger pelvic tumors, massive ascites, hyperviscosity syndrome, chemotherapy, and prolonged abdominal and pelvic surgeries in these patients; preoperative fasting and longer postoperative bed rest causing slow blood flow; and distant metastases, chemotherapy, and hormone therapy increasing postoperative thrombotic event risk [18].

Thrombosis affects ovarian cancer patients' prognosis, prolongs hospitalization, and increases economic burden. Postoperative venous thrombosis usually manifests as limb swelling and pain, but asymptomatic cases lead to missed diagnoses. The generalized G‐Caprini score assesses postoperative VTE risk in surgical patients, guiding anticoagulant timing and dosage. However, gynecologic oncology patients, especially those with ovarian cancer, have a higher VTE incidence compared to other cancers, with previous studies reporting 9.02%–32.6% postoperative thrombotic event incidence after ovarian cancer [18, 19, 20, 21, 22, 23, 24]. The traditional G‐Caprini score's validity in gynecologic oncology is less studied, and its reliability in assessing postoperative thrombotic events in ovarian cancer patients is controversial. With analytical method development, mathematical models based on multiple markers are increasingly used medically, but no perfect postoperative thrombosis risk assessment method exists for ovarian cancer patients. Studies show inflammation closely relates to VTE development, with patients exhibiting strong inflammatory responses prone to secondary thrombosis [10, 11, 25, 26, 27, 28]. Currently, no recognized single inflammatory marker assesses inflammatory response at VTE onset and VTE risk with inflammation. Therefore, designing a feasible, simple method accurately identifying high‐risk individuals for postoperative VTE after ovarian cancer surgery is important. Meanwhile, the Caprini score is typically applied preoperatively. Because our model incorporates postoperative biomarkers, direct “superiority” claims versus Caprini‐based models are not appropriate. Instead, these approaches are complementary: preoperative tools guide baseline prophylaxis, whereas the proposed score supports early postoperative reassessment. In external validation, performance was comparable with overlapping confidence intervals, highlighting the need for further multicenter validation.

Rationale for “counter‐intuitive” AST and PLT directions: Lower postoperative platelet counts may reflect perioperative platelet activation and consumption, hemodilution, transfusion effects, or redistribution during systemic inflammatory response rather than baseline thrombopoiesis. Similarly, the association between low AST and VTE risk should be interpreted cautiously; AST may behave as a surrogate marker of perioperative metabolic patterns rather than a causal determinant. These observations require confirmation in independent cohorts before mechanistic conclusions are drawn.

We selected the most significant indexes (Age, BMI, AST, INR, CRP, PLT) based on multivariate regression β‐coefficients to construct a predictive model. These include patients' basic clinical characteristics, liver function, coagulation function, and inflammatory indicators within 24 h postoperatively, better assessing ovarian cancer patients' systemic condition after surgery. Accessible in most hospitals, even primary ones, the total cost (186RMB) is affordable for most hospitalized patients. Importantly, we converted the diagnostic model into a scoring system facilitating clinical use, exhibiting good diagnostic performance in the training set and two validation sets.

This study integrated 40 parameters, including original metrics and ratio metrics reported in other studies, such as NLR, PLR, LMR. Recent studies reported inflammatory factors' involvement in VTE and predictive ability for VTE mortality risk [25, 29, 30]. In our study, leukocyte counts up to 24 h postoperatively (*p* < 0.001) and postoperative CRP values (*p* = 0.438) differed statistically between VTE and N‐VTE groups. After multivariate analysis in the training set, postoperative CRP and PLT were independent risk factors for postoperative thrombotic events, included in the prediction model. The AUC increased from 0.873 to 0.954 in the training set, 0.898 to 0.925 in internal validation upon including these inflammatory factors. Using data from another third‐class hospital as an external validation set tested the predictive model's diagnostic efficacy, similarly increasing the AUC from 0.779 to 0.809, indicating inflammatory factor inclusion enhanced the model's diagnostic efficacy.

Other scholars constructed VTE diagnostic models based on different clinical characteristics, achieving acceptable diagnostic performance. However, this retrospective study lacked external validation using other datasets. One study developed a predictive model applicable to VTE risk prediction after anticancer therapy initiation in common solid tumor patients [31]. However, some ovarian cancer patients experienced thrombotic events before initial therapy [32]. Our study considered common clinical indicators, tumor markers like CA125 and HE4, intraoperative ovarian cancer surgery conditions (duration, bleeding), and tumor factors (maximum diameter, ascites). Although postoperative pathology was not a predictive model parameter, pathology type significantly differed between VTE and N‐VTE groups (*p* < 0.001), with clear cell carcinoma proportion significantly higher in the VTE group (30.23% vs. 13.33%), aligning with conclusions that ovarian clear cell carcinoma patients are more likely to experience postoperative thrombosis [33]. Additionally, we proposed a new scoring system based on the diagnostic model. The AUC changes between the diagnostic nomogram and novel scoring system were small in the training and two validation sets, suggesting the nomogram‐derived scoring system is not only easy to use but also robustly diagnostically significant.

Model discrimination decreased in the external cohort (AUC 0.809 for the expanded model and 0.791 for the simplified score). The substantial decrease in AUC from the training cohort to the external cohort indicates model optimism and potential overfitting despite penalization. This finding reinforces the need for prospective validation and possible model recalibration before clinical deployment. Possible reasons include differences in patient case‐mix, imaging thresholds, and diagnostic intensity for VTE, perioperative prophylaxis practices, laboratory assay variation, and relatively small external event numbers. Although the events‐per‐variable ratio in the training cohort was approximately 5, which is lower than traditional recommendations, penalized regression with shrinkage and bootstrap optimism correction was applied to reduce overfitting; nevertheless, further validation in larger prospective cohorts is warranted. Limitations include the retrospective design, potential residual confounding, single‐region external validation, and limited event counts. Additionally, cutoffs were data‐derived and should be tested for stability across settings; future work should include bootstrap optimism correction and prospective multicenter validation. Both cohorts were derived from tertiary centers within the same university system and geographic region, which may limit transportability to other healthcare settings with different prophylaxis protocols or diagnostic practices. Cutoff values were derived using the Youden index and should be interpreted as statistically optimized thresholds rather than clinically prespecified decision thresholds. Future studies should evaluate clinically anchored thresholds based on prophylaxis decision points and bleeding risk.

In conclusion, we developed and externally validated an early postoperative risk score for VTE in epithelial ovarian cancer. Although discrimination was high in development cohorts and acceptable in external validation, the observed optimism underscores the need for prospective multicenter validation and potential recalibration. The proposed score is intended to complement preoperative tools by supporting early postoperative reassessment and targeted surveillance rather than replacing established prophylaxis models.

## Ethics Statement

Declaration of Helsinki, with the approval of the Ethical Committee of The First Affiliated Hospital of Sun Yat‐sen University (No. S/55904). Since this study was retrospective, involving no risk for the participants, most patients were dead when this analysis was performed and contacting the families could cause unnecessary suffering; informed consent requirements from the subjects were waived.

## Conflicts of Interest

The authors declare no conflicts of interest.

## Data Availability

Data is available from the corresponding author on request.
